# Ascending to Q1: A critical analysis of clinics journal's editorial trajectory and future challenges

**DOI:** 10.1016/j.clinsp.2025.100722

**Published:** 2025-08-04

**Authors:** José Maria Soares-Júnior, Fauze Maluf-Filho

**Affiliations:** Faculdade de Medicina da Universidade de São Paulo, São Paulo, Brazil

*Clinics* journal plays a pivotal role in disseminating high-quality medical and biomedical research, serving as a bridge between scientific innovation and clinical practice. Since its inception, the journal has been dedicated to publishing original contributions and review articles from over 50 countries, fostering a global exchange of knowledge. Its open-access model ensures that cutting-edge research is accessible to clinicians, researchers, and policymakers worldwide, thereby accelerating the translation of scientific discoveries into real-world healthcare solutions. By covering a wide range of medical specialties, *Clinics* addresses diverse health challenges, making it an invaluable resource for the medical community^1^.

The recent announcement of the 2024 Impact Factor (IF) scores marks a significant milestone for *Clinics*, as the journal has achieved an IF of 2.4, ascending to Q1 in the *Medicine, General and Internal* category. This accomplishment reflects a steady upward trajectory, with a 0.2-point increase from the 2023 IF and a notable leap from Q2 to Q1, advancing from position 93 to 78 out of 332 journals in the category. Such progress underscores the dedication of the editorial team, contributors, and collaborators in elevating the journal's academic rigor and global relevance^2^.

The transition to Q1 is a testament to the collective efforts of Hospital das Clínicas, and the broader team, whose commitment to excellence aligns with Elsevier's strategic vision. This achievement not only enhances the journal's reputation but also strengthens its capacity to attract high-quality research, fostering innovation in clinical and internal medicine. These metrics reflect the journal’s commitment to maintaining rigorous editorial standards and publishing research of significant scientific relevance. The partnership with Elsevier has further enhanced Clinics’ capabilities, streamlining the submission and review processes to ensure a timely publication of high-quality manuscripts. This collaboration not only bolsters the journal’s international reputation but also amplifies its reach, attracting submissions from leading researchers across the globe^3^.

As we celebrate this milestone, it is imperative to maintain momentum. The inclusion of impact factor trends and journal rankings in this announcement serves as a roadmap for future goals. To sustain and build upon Q1 status, *Clinics* must continue prioritizing cutting-edge research, robust peer review, and interdisciplinary engagement. We invite the scientific community to contribute to this journey, ensuring the journal remains a beacon of knowledge and progress in its field^1^^,^^4^.

Beyond its academic contributions, *Clinics* serves as a platform for fostering international collaboration and inclusivity in medical research. With an Editorial Board representing 13 countries, the journal embodies a commitment to diverse perspectives and equitable representation in scientific discourse. By continuously expanding its network of Section Editors, reviewers, and authors, *Clinics* strengthens its mission to address global health issues and promote evidence-based medicine^5^. As the journal enters this new era under Elsevier’s stewardship, its role in shaping the future of medical research and practice remains more critical than ever.

To build on its Q1 achievement and further elevate its global standing, *Clinics* should implement targeted strategies such as launching themed issues on emerging topics like Artificial Intelligence and Robotics in medicine, global health disparities, or post-pandemic healthcare innovations, mainly in genetic engineering and mathematics. These special editions, curated with international experts, would attract high-impact research and highlight interdisciplinary collaboration. Additionally, fostering cross-institutional partnerships with leading universities and research hubs—particularly in underrepresented regions — could diversify authorship and broaden the journal’s scope^6^. Finally, hosting annual virtual symposia or author workshops would strengthen community engagement, aligning with *Clinics*’ mission to bridge research and clinical practice. These actionable steps would not only sustain momentum but also reinforce the journal’s role as a catalyst for transformative medical science.

## Declaration of competing interest

The authors declare no conflicts of interest.
